# Neutrophil-to-lymphocyte ratio (NLR), platelet-to-lymphocyte ratio (PLR), and systemic immune inflammation index (SII) to predict postoperative pneumonia in elderly hip fracture patients

**DOI:** 10.1186/s13018-023-04157-x

**Published:** 2023-09-12

**Authors:** Wei Yao, Wei Wang, Wanyun Tang, Qiaomei Lv, Wenbo Ding

**Affiliations:** 1 Department of Orthopedics, Dandong Central Hospital, China Medical University, No. 338 Jinshan Street, Zhenxing District, Dandong, 118002 Liaoning Province People’s Republic of China; 2 Department of Oncology, Dandong Central Hospital, China Medical University, Dandong, China

**Keywords:** Hip fracture, NLR, PLR, SII, Predictive value, Postoperative pneumonia

## Abstract

**Purpose:**

Investigate the association between the neutrophil-to-lymphocyte ratio (NLR), platelet-to-lymphocyte ratio (PLR), and systemic immune-inflammation index (SII) about the presence of postoperative pneumonia (POP) in geriatric patients with hip fractures. Compare the predictive value of these biomarkers for POP and assess their potential for early detection of POP.

**Methods:**

We retrospectively included elderly patients with hip fractures who underwent surgical treatment at our institution. POP was diagnosed according to the guidelines provided by the American Thoracic Society. We collected neutrophil, lymphocyte, and platelet counts upon admission to calculate the NLR, PLR, and SII. Receiver operating characteristic curves were utilized to establish the optimal cutoff values for each index. Multivariate logistic regression analysis and propensity score matching analysis were utilized to assess the independent association between each index and POP after adjusting for demographic, comorbidity, and surgery-related variables.

**Results:**

The study included a total of 1199 patients, among whom 111 cases (9.26%) developed POP. NLR exhibited the highest predictive value for POP in elderly patients with hip fractures compared to PLR and SII (AUC = 0.648, 95% CI 0.594–0.701). A high NLR, using the optimal cutoff value of 5.84, was significantly associated with an increased incidence of POP (OR = 2.24, 95% CI 1.43–3.51). This finding remained statistically significant even after propensity score matching (OR = 2.04, 95% CI 1.31–3.20).

**Conclusions:**

Among the three inflammatory/immune markers considered, the NLR demonstrates the highest reliability as a predictor for POP in elderly patients with hip fractures. Therefore, it serves as a valuable tool for early identification.

**Supplementary Information:**

The online version contains supplementary material available at 10.1186/s13018-023-04157-x.

## Introduction

Hip fractures pose a significant health concern particularly within the elderly community, given its escalating incidence and substantial socio-economic impact [1–3]. Older individuals are predisposed to complications, such as osteoporosis and sarcopenia, potentially leading to an amplified risk of fractures and delayed postoperative recovery [4, 5]. Key to the management of hip fractures is the prompt execution of surgical intervention, ideally within the first 24 h following the injury [1, 6, 7]. Existing data suggest that efficient management of hip fractures, especially through early surgery and the involvement of geriatric orthopedic specialists, enhances a patient’s prognosis [8–11]. Notwithstanding, perioperative complications continue to present a considerable challenge [11].

Postoperative pneumonia (POP) is a prevalent complication among elderly patients with hip fractures, with an incidence ranging from 5.1 to 14.9% [12–14]. Despite the implementation of strategies, such as antibiotic therapy, promotion of coughing and sputum clearance after surgery, and perioperative pulmonary rehabilitation training, the occurrence of pulmonary infection remains relatively high [15, 16]. This hampers postoperative recovery, extends hospitalization duration, elevates medical expenses, and even contributes to potential fatalities [17, 18]. The systemic inflammatory response contributes to the secondary injury associated with hip fractures and is correlated with the development of POP [19, 20]. Lung organ damage mediated by neutrophils and subsequent pulmonary infection are the primary causes of mortality in elderly patients with hip fractures [21]. The diminished physiological reserves in older patients make them more susceptible to the release of inflammatory cytokines after injury while lacking adequate anti-inflammatory mediators to counterbalance [22]. Recent years have witnessed a substantial advancement in comprehending systemic inflammatory response and lung injury pathophysiology in elderly hip fracture patients [23, 24]. Fractures and surgical procedures promptly trigger a sterile systemic inflammatory response and lung injury, thereby augmenting the likelihood of POP occurrence [25]. Thus, evaluating the inflammatory status aids in predicting the occurrence of POP in hip fracture patients.

The NLR serves as a marker for subclinical inflammation, reflecting the innate immune response mediated by neutrophils and the adaptive immune response supported by lymphocytes [26]. Previous studies have demonstrated NLR's superiority over traditional blood inflammatory markers in predicting stroke-associated pneumonia (SAP) and community-acquired pneumonia (CAP) [27, 28]. The PLR, on the other hand, is a marker associated with platelet aggregation and systemic inflammation, thereby enabling the evaluation of inflammatory coagulation reactions and platelet activation resulting from systemic inflammatory response [29]. Furthermore, alterations in certain inflammatory/immune markers (NLR and PLR) have been observed after long bone fractures [30]. The SII is a novel marker that combines peripheral blood lymphocyte, neutrophil, and platelet counts and is linked to inflammation [31]. Previous studies have highlighted SII's high prognostic value in cancer patients, as well as its significance in predicting POP in lung cancer patients [32, 33]. However, the association between these three inflammatory/immune markers and the development of POP in fracture patients remains uncertain. To the best of our knowledge, no studies have explored the relationship between inflammatory/immune markers and the occurrence of POP in patients with hip fractures. Therefore, this study aims to determine the optimal cutoff values for NLR, PLR, and SII, evaluate the ability of NLR, PLR, and SII levels at admission to predict POP and investigate the independent connections of these three markers with POP.

## Materials and methods

This retrospective cohort study was conducted at a level-one trauma center from March 2014 to March 2023. The study enrolled patients aged 60 and above who had acute hip fractures and received orthopedic surgical treatment. The inclusion criteria specified patients who met these age and fracture requirements, while the exclusion criteria excluded patients with old fractures (injury to surgery time greater than 3 weeks), pathologic fractures, multiple fractures, periprosthetic fractures, or open fractures. Patients with a history of conservative treatment, revision surgery, reoperation, or long-term use of immunosuppressive agents such as glucocorticoids were also excluded. Additionally, patients who had pneumonia or respiratory tract inflammation preoperatively, patients who died during hospitalization, and patients with incomplete data for any reason were excluded. The study was approved by the hospital ethics review committee and adheres to the STROBE guidelines and the Helsinki Declaration. As the data used in this study are retrospective and anonymous, the ethics committee granted exempt consent for this cohort study. More details can be found in the flowchart (Additional file 1: eFigure 1).

### Exposure

Biomarkers were collected through hematological tests within 24 h of hospital admission in patients diagnosed with hip fractures [34]. The collected data encompassed counts for neutrophils, lymphocytes, and platelets. The NLR, PLR, and SII metrics were calculated using the respective formulas: NLR = neutrophil count/lymphocyte count; PLR = platelet count/lymphocyte count; and SII = (neutrophil count × platelet count)/lymphocyte count [26, 35, 36].

### Outcome

Postoperative pneumonia (POP) is defined as the emergence of new infiltrates on postoperative chest X-rays or chest computed tomography (CT), which are confirmed through consultation with a respiratory physician [17]. The diagnosis of POP relies on specific criteria outlined in the American Thoracic Society guidelines for healthcare-associated pneumonia [37]: (1) the presence of new and/or progressive and persistent respiratory symptoms, such as cough and purulent secretions; (2) postoperative fever (body temperature > 38.0 °C) or hypothermia (body temperature < 36.0 °C); (3) physical examinations revealing lung consolidation and auscultatory crackles; (4) white blood cell count that exceeds 10 × 109/L or falls below 4 × 109/L; and (5) identification of patchy inflammatory shadows or interstitial changes on chest X-rays. If a patient satisfies any of the criteria from 1 to 4 in conjunction with criterion 5, a diagnosis of POP can be established, provided that lung cancer, tuberculosis, pulmonary embolism, and other pulmonary diseases have been ruled out. POP is assessed as the primary outcome measure from the initial 24 h following surgery until discharge.

### Covariables

Based on the relevant literature [38–40] and patient information obtained from our institution, we extracted demographic characteristics, comorbidity data, and surgery-related information from electronic medical records. Demographic characteristics encompassed age, gender, smoking, and drinking status. Comorbidity data included hypertension, diabetes, chronic obstructive pulmonary disease (COPD), cardiovascular disease, stroke, chronic liver disease, chronic kidney disease, and tumors. Surgery-related information consisted of the type of hip fracture (femoral neck, intertrochanteric, subtrochanteric), American Society of Anesthesiologists (ASA) classification, surgical procedure (total hip arthroplasty, hemiarthroplasty, intramedullary nail, plate, and screw fixation), intraoperative blood loss, postoperative intensive care unit monitoring, duration of surgery, and bed rest time. Prior to commencing the study, all researchers underwent professional training, and to minimize potential errors, all data were entered twice and cross-checked, with any discrepancies being resolved through consensus discussions.

### Statistical analysis

Continuous variables were reported as mean ± standard deviation (SD), and categorical variables were presented as frequency and percentage (%). A two-sided *p*-value of < 0.05 was considered statistically significant. The diagnostic performance of six indicators (neutrophils, lymphocytes, platelets, NLR, PLR, and SII) was assessed using the receiver operating characteristic (ROC) curve. The diagnostic performance was compared by calculating the area under the ROC curve (AUC) and conducting a comparison of AUCs using the DeLong test [41]. The optimal cutoff values for the six indicators were determined by maximizing the Youden index (sensitivity + specificity—1). The performance of these indicators in predicting POP was assessed by evaluating sensitivity, specificity, positive predictive value (PPV), negative predictive value (NPV), and their respective 95% confidence intervals (CIs).

NLR, PLR, and SII were grouped using predetermined optimal cutoff values and then analyzed through multivariable logistic regression, adjusting for several potential confounding factors (*p* > 0.1), to investigate their association with POP. Initially, univariable regression analysis was conducted, and variables with a *p*-value < 0.10 were included in the subsequent multivariable logistic regression analysis. Propensity score matching (PSM) was performed to minimize potential confounding effects between groups and adjust for covariates. The nearest neighbor matching algorithm with a caliper width of 0.25 standard deviations (SD) was used for propensity score matching analysis [42]. Covariates were matched in a 1:1 ratio between the two groups. The characteristics between the two groups were assessed using the standardized mean difference (SMD). Additionally, patients were divided into four groups (Q1, Q2, Q3, and Q4) based on the quartile distribution of NLR, PLR, and SII levels. This categorization enabled a more accurate assessment of the dose–response relationship between the three markers and POP.

Subgroup analysis was performed in the propensity score matching (PSM) cohort to further explore the diagnostic value of NLR and SII. The PSM cohort was stratified into multiple subgroups based on all covariates, and univariable logistic regression analysis was performed to determine the odds ratio (OR) and 95% confidence interval (CI) for postoperative pneumonia (POP) associated with high NLR and high SII. The relationship between subgroups was assessed, and statistical significance was defined as a *p*-value of less than 0.01 to account for multiple subgroups [43]. Statistical analyses were performed using GraphPad Prism 9.0 and R version 4.2.0.

## Results

**Fig. 1:**
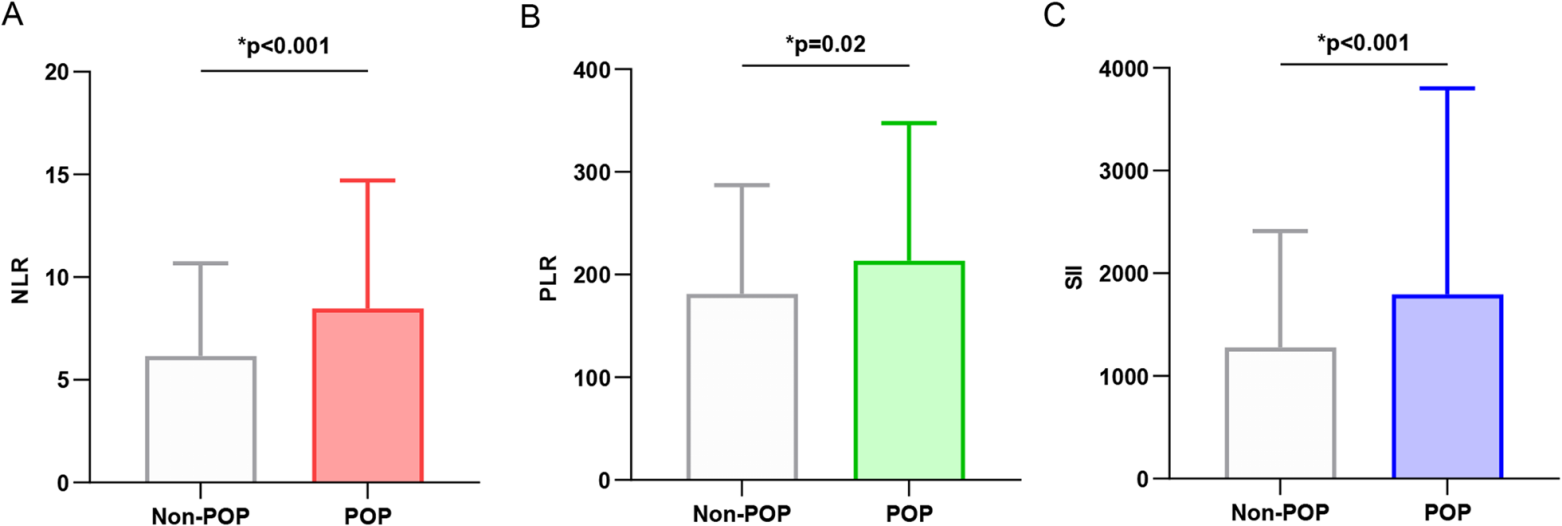
The bar graph displays the distribution of NLR, PLR, and SII values between the non-POP group (*n* = 1008) and the POP group (*n* = 111). **a** The NLR values in the POP group were higher than in the non-POP group (*p* < 0.001). **b** The PLR values in the POP group were higher than in the non-POP group (*p* = 0.02). **c** The SII values in the POP group were higher than in the non-POP group (*p* < 0.001). NLR refers to neutrophil-to-lymphocyte ratio; PLR refers to platelet-to-lymphocyte ratio; SII refers to systemic immune-inflammation index; POP refers to postoperative pneumonia; Non-POP refers to non-postoperative pneumonia

This study involved a total of 1199 elderly patients diagnosed with hip fractures, with an average age of 74.77 years. Out of the participants, 720 were female (60.05%). Among the sample, 111 patients (9.26%) experienced postoperative pneumonia (POP). Table 1 presents the baseline characteristics of elderly hip fracture patients. In comparison to the non-POP group, the POP group exhibited advanced age (*p* < 0.001), higher ASA classification (*p* < 0.001), longer duration of bed rest (*p* < 0.001), increased prevalence of pre-existing complications, and a higher rate of postoperative ICU admission (*p* < 0.001). Moreover, there was a significantly higher incidence of POP among patients with intertrochanteric fractures than those with femoral neck and proximal femur fractures (*p* < 0.001). Additionally, patients who underwent intramedullary nail fixation surgery had a higher POP occurrence than other surgical methods (*p* = 0.003). The NLR (*p* < 0.001), PLR (*p* = 0.02), and SII (*p* < 0.001) values were all significantly elevated in the POP group, as illustrated in Table 1 and Fig. 1.

**Table 1:** Baseline characteristics of the 1199 patients with hip fractures

Characteristics	Total (*n* = 1199)	POP development		
		Non-POP (*n* = 1088)	POP (*n* = 111)	*p*-value
*Demographic*				
Age, × years (Mean, SD)	74.77 (9.61)	74.17 (9.56)	80.68 (8.04)	< 0.001
Female gender (*n*, %)	720 (60.05)	653 (60.02%)	67 (60.36%)	0.94
Smoking (*n*, %)	205 (17.10)	186 (17.10%)	19 (17.12%)	0.99
Alcohol (*n*, %)	136 (11.34)	123 (11.31%)	13 (11.71%)	0.90
*Comorbidities*				
Hypertension (*n*, %)	601 (50.13)	537 (49.36%)	64 (57.66%)	0.09
Diabetes (*n*, %)	275 (22.94)	243 (22.33%)	32 (28.83%)	0.12
COPD (*n*, %)	137 (11.43)	92 (8.46%)	45 (40.54%)	< 0.001
Cardiovascular disease (*n*, %)	372 (31.03)	323 (29.69%)	49 (44.14%)	0.002
Stroke (*n*, %)	310 (25.85)	261 (23.99%)	49 (44.14%)	< 0.001
Chronic liver disease (*n*, %)	53 (4.42)	43 (3.95%)	10 (9.01%)	0.01
Chronic kidney disease (*n*, %)	62 (5.17)	50 (4.60%)	12 (10.81%)	0.01
Neoplasms (*n*, %)	115 (9.59)	98 (9.01%)	17 (15.32%)	0.03
*Operation*				
*Fracture type*				
Femoral neck fracture (*n*, %)	613 (51.13)	574 (52.76%)	39 (35.41%)	< 0.001
Intertrochanteric fracture (*n*, %)	514 (42.87)	445 (40.90%)	69 (62.16%)	
Subtrochanteric fracture (*n*, %)	72 (6.01)	69 (6.34%)	3 (2.70%)	
*ASA classification*				
I–II (*n*, %)	539 (44.95)	513 (47.15%)	26 (23.42%)	< 0.001
III–V (*n*, %)	660 (55.05)	575 (52.85%)	85 (76.58%)	
*Surgery method*				
Total Hip Arthroplasty (*n*, %)	148 (12.34)	140 (12.87%)	8 (7.21%)	0.003
Hemiarthroplasty (*n*, %)	285 (23.76)	259 (23.81%)	26 (23.42%)	
Intramedullary nail fixation (*n*, %)	409 (34.11)	357 (32.81%)	52 (46.85%)	
Internal fixation with steel plate (*n*, %)	165 (13.76)	147 (13.51%)	18 (16.22%)	
Internal fixation with hollow nails (*n*, %)	192 (16.01)	185 (17.00%)	7 (6.31%)	
Intraoperative blood loss, × ml (Mean, SD)	178.11 (153.87)	177.00 (155.64)	189.03 (135.51)	0.10
Postoperative ICU care (*n*, %)	57 (4.75)	40 (3.68%)	17 (15.32%)	< 0.001
Operation time, × hours (Mean, SD)	1.67 (0.81)	1.67 (0.82)	1.72 (0.74)	0.14
Bedridden time, × days (Mean, SD)	5.90 (3.99)	5.68 (3.81)	8.02 (4.97)	< 0.001
*Laboratory findings*				
NEU count, × 10^9/L (Mean, SD)	6.82 (2.81)	6.71 (2.72)	7.91 (3.41)	< 0.001
LYM count, × 10^9/L (Mean, SD)	1.32 (0.58)	1.34 (0.59)	1.14 (0.50)	< 0.001
PLT, × 10^9/L (Mean, SD)	208.57 (81.22)	208.86 (79.92)	205.72 (93.42)	0.20
NLR^a^ (Mean, SD)	6.38 (4.74)	6.16 (4.51)	8.47 (6.23)	< 0.001
PLR^b^ (Mean, SD)	184.13 (109.18)	181.11 (105.97)	213.66 (133.77)	0.02
SII^c^ (Mean, SD)	1326.03 (1249.27)	1278.25 (1134.95)	1794.33 (2006.10)	< 0.001

POP, Postoperative pneumonia; COPD, Chronic obstructive pulmonary disease; ASA, The American Society of Anesthesiologists Physical Status Classification System; ICU, Intensive Care Unit; NEU, Neutrophil; LYM, Lymphocyte; PLT, Platelet

^a^NLR = Neutrophil count/Lymphocyte count

^b^PLR = Platelet count/Lymphocyte count

^c^SII = (Neutrophil count × Platelet count)/Lymphocyte count

**Fig. 2:**
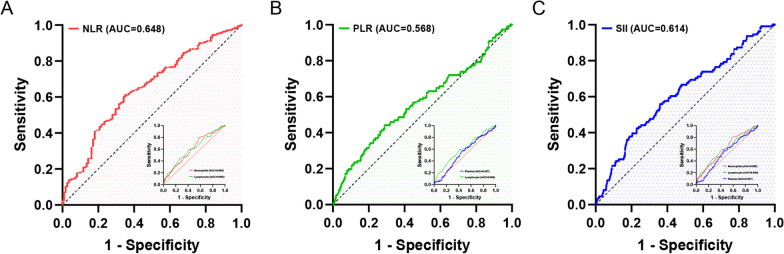
ROC curve analysis was performed to evaluate the predictive value of each immune/inflammatory marker for postoperative pneumonia in hip fracture patients. Compared to the established scoring system for hip fracture patients, which includes PLR and SII **b**, **c**, NLR (**a**) demonstrated the highest accuracy in predicting postoperative pneumonia. ROC refers to receiver operating characteristic; AUC represents the area under the curve; NLR refers to neutrophil-to-lymphocyte ratio; PLR refers to platelet-to-lymphocyte ratio; SII refers to systemic immune-inflammation index

ROC analysis was conducted to assess the predictive capacity of biomarkers for POP in hip fracture patients (Fig. 2). Based on the AUC values (Additional file 1: eFigure 2), NLR (AUC = 0.648, 95% CI 0.594–0.701) and SII (AUC = 0.614, 95% CI 0.558–0.629) exhibited superior predictive abilities compared to PLR (AUC = 0.568, 95% CI 0.507–0.629). Notably, NLR demonstrated the highest predictive value among the considered biomarkers (DeLong’s test: all *p*-values < 0.05, Table 2). Refer to Table 2 for detailed insights into the optimal cutoff points, specificity, sensitivity, and other relevant information.

**Table 2:** Assessment of the characteristic parameters of each biomarker

Variables	Cutoff point	AUC (95% CI)	SEN (%, 95% CI)	SPE (%, 95% CI)	PPV (%, 95% CI)	NPV (%, 95% CI)	DeLong test^d^ (*p*-value)
NLR^a^	5.84	0.648 (0.594–0.701)	65.8 (56.9–74.6)	60.3 (57.4–63.2)	14.5 (11.4–17.5)	94.5 (92.8–96.2)	Reference
PLR^b^	204.41	0.568 (0.507–0.629)	44.1 (34.9–53.4)	71.1 (68.4–73.8)	13.5 (10.0–17.0)	92.6 (90.8–94.4)	0.003
SII^c^	1243.07	0.614 (0.558–0.671)	55.9 (46.6–65.1)	64.2 (61.3–67.0)	13.7 (10.5–16.9)	93.4 (91.7–95.2)	0.042
NEU	5.85	0.605 (0.551–0.660)	79.3 (71.7–86.8)	42.7 (39.8–45.7)	12.4 (10.0–14.8)	95.3 (93.4–97.2)	0.049
LYM	1.12	0.604 (0.548–0.661)	59.5 (50.3–68.6)	58.2 (55.2–61.1)	12.7 (9.80–15.5)	93.4 (91.5–95.2)	0.037
PLT	187.50	0.537 (0.482–0.592)	56.8 (47.5–66.0)	55.1 (52.1–58.0)	11.4 (8.80–14.1)	92.6 (90.6–94.6)	0.006

NEU, Neutrophils; LYM, Lymphocyte; PLT, Platelet; CI, confidence interval; AUC, the area under the curve; SEN, Sensitivity; SPE, Specificity; PPV, positive predictive value; NPV, negative predictive value

^a^NLR = Neutrophil count/Lymphocyte count

^b^PLR = Platelet count/Lymphocyte count

^c^SII = (Neutrophil count × Platelet count)/Lymphocyte count

^d^A comparison of AUC was performed using the DeLong test

To investigate the relationship between NLR, PLR, and SII levels in elderly hip fracture patients and the occurrence of POP, a thorough evaluation was conducted, and the comprehensive results are presented in Table 3. According to the optimal cutoff values, in the univariable regression analysis, elevated levels of NLR (OR = 2.92, 95% CI 1.94–4.40) and SII (OR = 2.27, 95% CI 1.53–3.36) exhibited a significant correlation with POP. Even after controlling for potential confounding factors, a significant association between NLR levels (OR = 2.24, 95% CI 1.43–3.51) and SII levels (OR = 1.76, 95% CI 1.14–2.73) and POP was observed. It is worth noting that there was no significant association between an increase in PLR levels, after controlling for potential confounding factors, and the occurrence of POP (OR = 1.37, 95% CI 0.88–2.15). Detailed results of the multivariable regression analysis can be found in the Additional file 1: eTables 1–3.

**Table 3:** Unadjusted and adjusted associations between postoperative pneumonia and inflammation/immunity markers based on different cut-off values

Biomarker	Group		Unadjusted OR (95% CI)	*P*	Multivariable regression adjusted OR (95% CI)	*P*	PSM adjusted OR (95% CI)	*P*
NLR^a^	Continuous	Per SD	1.40 (1.21–1.62)	< 0.001	1.20 (1.01–1.42)	0.04	NA	NA
	Best cutoff^d^	< 5.84	1 [Reference]	< 0.001	1 [Reference]	< 0.001	1 [Reference]	0.002
		≥ 5.84	2.92 (1.94–4.40)		2.24 (1.43–3.51)		2.04 (1.31–3.20)	
	Quartile	Q1 (< 3.53)	1 [Reference]	< 0.001*	1 [Reference]	0.01*	1 [Reference]	0.02*
		Q2 (3.53–5.29)	1.03 (0.51–2.08)		0.73 (0.34–1.55)		0.40 (0.18–0.89)	
		Q3 (5.29–7.75)	2.22 (1.20–4.12)		1.80 (0.92–3.52)		1.34 (0.79–2.27)	
		Q4 (> 7.75)	3.02 (1.66–5.49)		1.76 (0.92–3.37)		1.78 (1.06–2.98)	
PLR^b^	Continuous	Per SD	1.25 (1.08–1.46)	0.004	1.13 (0.94–1.36)	0.20	NA	NA
	Best cutoff^@^	< 204.41	1 [Reference]	0.001	1 [Reference]	0.16	1 [Reference]	0.10
		≥ 204.41	1.95 (1.31–2.90)		1.37 (0.88–2.15)		1.50 (0.93–2.42)	
	Quartile	Q1 (< 115.63)	1 [Reference]	0.04*	1 [Reference]	0.54*	1 [Reference]	0.74*
		Q2 (115.63–156.25)	0.68 (0.37–1.25)		0.72 (0.37–1.41)		0.46 (0.24–0.89)	
		Q3 (156.25–226.67)	0.88 (0.49–1.56)		0.83 (0.44–1.56)		0.68 (0.39–1.21)	
		Q4 (> 226.67)	1.60 (0.96–2.68)		1.12 (0.63–2.02)		1.30 (0.80–2.10)	
SII^c^	Continuous	Per SD	1.31 (1.13–1.51)	< 0.001	1.19 (1.00–1.43)	0.05	NA	NA
	Best cutoff^d^	< 1243.07	1 [Reference]	< 0.001	1 [Reference]	0.01	1 [Reference]	0.01
		≥ 1243.07	2.27 (1.53–3.36)		1.76 (1.14–2.73)		1.84 (1.17–2.88)	
	Quartile	Q1 (< 635.08)	1 [Reference]	< 0.001*	1 [Reference]	0.01*	1 [Reference]	0.02*
		Q2 (635.08–993.83)	0.74 (0.38–1.45)		0.70 (0.34–1.46)		0.65 (0.34–1.23)	
		Q3 (993.83–1645.41)	1.31 (0.72–2.37)		1.23 (0.64–2.36)		1.09 (0.64–1.85)	
		Q4 (> 1645.41)	2.47 (1.44–4.25)		1.97 (1.07–3.63)		1.88 (1.16–3.04)	

^a^NLR = Neutrophil count/Lymphocyte count

^b^PLR = Platelet count/Lymphocyte count

^c^SII = (Neutrophil count × Platelet count)/Lymphocyte count; SD, standard deviation; NA, not available; OR, odds ratio; PSM, propensity scores matching; * *p* for trend

^d^The best cutoff values for NLR, PLR, and SII were determined using Youden’s index

The optimal cutoff values for NLR, PLR, and SII were utilized to perform propensity score matching on various patient covariates. This method helped to minimize the influence of confounding factors between the groups. Following the matching process, all covariates were effectively balanced (SMD < 0.1, see Additional file 1: eTables 4–6). Remarkably, even after matching, there remained a significant relationship between elevated levels of NLR (OR = 2.04, 95% CI 1.31–3.20) and SII (OR = 1.84, 95% CI 1.17–2.88) and the occurrence of POP, thus strengthening the reliability and accuracy of our findings.

**Fig. 3:**
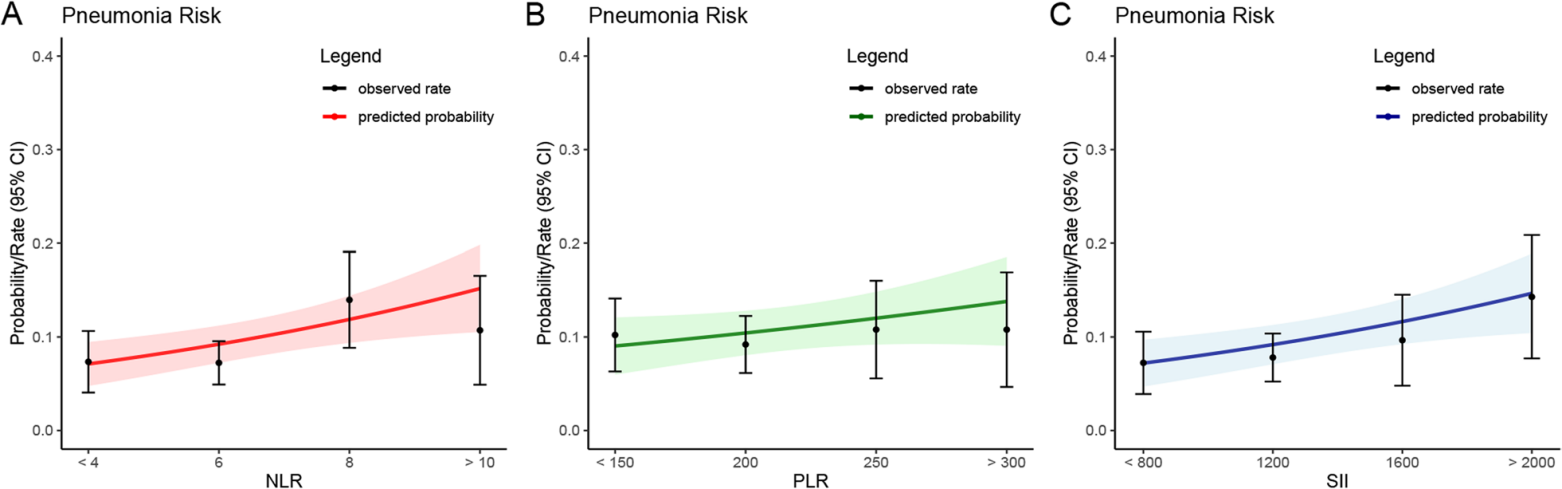
The relationship between immune/inflammatory markers and postoperative pneumonia in elderly patients with hip fractures was examined. Propensity score matching-adjusted odds ratios (ORs) and 95% confidence intervals (CIs) for postoperative pneumonia were estimated based on the baseline levels of NLR (**a**), PLR (**b**), and SII (**c**)

**Fig. 4:**
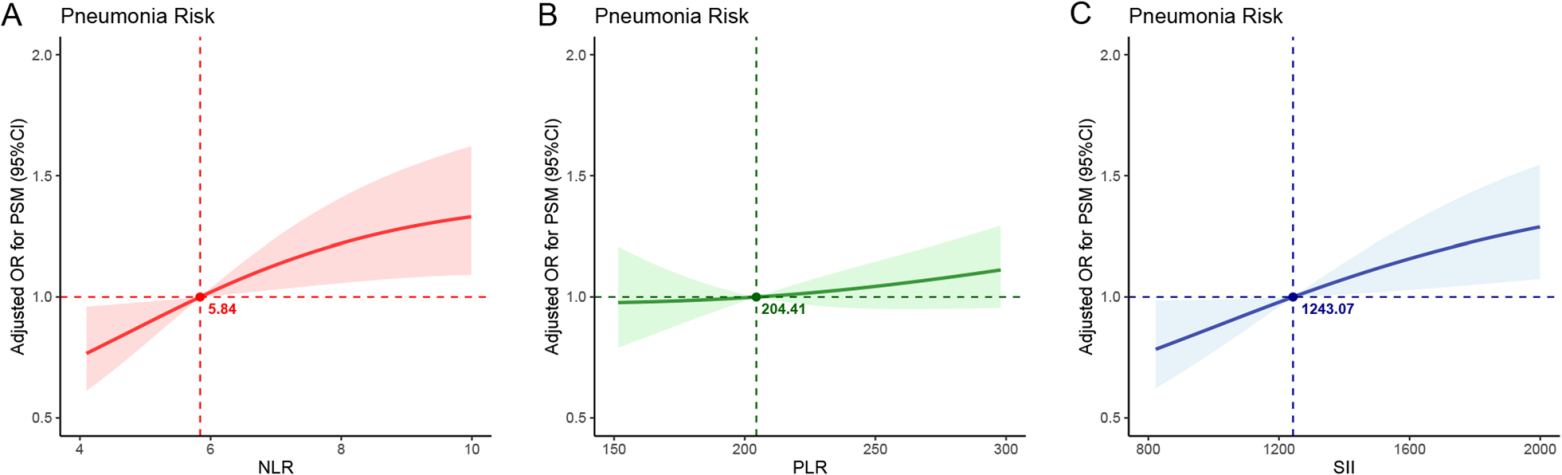
The relationship between immune/inflammatory markers and postoperative pneumonia in elderly patients with hip fractures was examined. The predictive probabilities and observed rates of postoperative pneumonia were analyzed based on the baseline levels of NLR (**a**), PLR (**b**), and SII (**c**)

When assessing NLR, PLR, and SII as continuous variables (refer to Table 3), we found that one standard deviation (SD) increase in NLR was associated with an adjusted odds ratio of 1.20 (95% CI 1.01–1.42) for POP. Similarly, a one-SD increase in PLR was linked to an adjusted OR of 1.13 (95% CI 0.94–1.36), and a one-SD increase in SII was associated with an adjusted OR of 1.19 (95% CI 1.00–1.43) for POP. Additionally, quartile analysis of NLR, PLR, and SII revealed explicit evidence of a dose–response relationship between NLR and SII with POP (*p* trend < 0.01). The summarized findings are presented in Table 3, and the association between elevated NLR and SII levels and a higher incidence of POP is visually depicted in Figs. 3 and 4.

Subgroup analyses were additionally performed on the covariates of NLR and SII, as presented in the Additional file 1: eFigures 3–4). These analyses demonstrated that there were no statistically significant interactions between NLR and SII with any of the covariates (all *p*-values for interactions ≥ 0.01).

## Discussion

The prevalence of POP in elderly patients with hip fractures exhibits variability across different institutions and regions [38, 44]. Nevertheless, the prevalence remains considerable and warrants attention. The diagnosis of POP poses challenges as traditional biomarkers such as white blood cells, procalcitonin (PCT), and C-reactive protein (CRP) demonstrate varied sensitivity and specificity in forecasting its occurrence [33, 45]. Moreover, chest X-ray examinations, an essential component in the diagnostic procedure, may not effectively identify infiltrations during preliminary stages of infection, instead functioning as a secondary measure in later stages [46, 47]. Regrettably, treatment delays could hinder patient recovery and exacerbate functional prognosis, highlighting the need for additional biomarkers with accurate predictive abilities for POP [28, 31]. In this study, the occurrence of POP in hip fracture patients was 9.26%, aligning with previous literature [14, 38, 48, 49]. We assessed the predictive ability of three inflammatory/immunological markers to determine their association with POP. The findings revealed that NLR and SII exhibit favorable predictive capabilities for POP and remain independent risk factors in adjusted analyses encompassing multivariable logistic regression and propensity score matching. Notably, our study underscores a significant statistical correlation between elevated NLR levels and increased susceptibility to POP when NLR exceeds the optimal cutoff value of 5.84. Among all tested inflammatory/immunological markers, NLR emerged as the most robust predictor for this condition. Consequently, it is recommended to calculate NLR and SII values, especially NLR, upon admission of elderly hip fracture patients to identify individuals who may benefit from intensified respiratory support and intervention targeting respiratory infections, thereby ensuring a favorable prognosis [26, 31, 48].

In recent years, there has been an increasing utilization of inflammatory/immune markers in major orthopedic surgeries, as they have shown associations with perioperative complications and long-term postoperative mortality rates [36, 50–53]. However, to the best of our knowledge, there have been no studies examining the relationship between inflammatory/immune markers and POP in elderly patients with hip fractures. Previous research has established connections between inflammatory/immune markers and both SAP and CAP [26, 28, 54–56]. Cheng et al. [54] conducted a comprehensive review of 734 stroke patients, revealing that an elevated neutrophil-to-lymphocyte ratio (NLR) exceeding the optimal cutoff value of 3.60 was independently associated with SAP (OR = 2.80, 95% CI 1.30–6.03). Through analyzing 5669 stroke patients, Xie et al. [55] discovered that higher levels of the SII could serve as a predictive factor for mortality risk in patients with SAP (HR = 2.06, 95% CI 1.26–3.38). Acar et al. [56] conducted a prospective study involving CAP patients, demonstrating the predictive value of SII levels for mortality rates in CAP patients. Further studies have also confirmed the diagnostic and predictive capabilities of NLR in CAP patients [26, 28].

The NLR serves as a marker of subclinical inflammation and demonstrates greater exacerbation potential in numerous pulmonary diseases [57, 58]. Patients with elevated NLR values tend to exhibit relatively lower lymphocyte counts and higher neutrophil counts, thereby providing an indirect assessment of both inflammation status and cell-mediated immunity [59]. The systemic immune-inflammation index (SII), a novel inflammation-related metric, has garnered attention due to its potential prognostic value in cancer patients [60]. A recent substantial retrospective study unveiled a significant correlation between elevated SII levels and the incidence of POP in cancer patients, effectively predicting the occurrence of POP [33].

The precise mechanisms underlying the association between inflammatory/immune markers and POP remain unclear. However, markers such as the NLR and SII have demonstrated a close correlation with systemic inflammatory response status and have predictive value for various infections, tumors, and autoimmune diseases [61, 62]. The potential mechanism may involve a cascade reaction of inflammatory cytokines and chemokines, triggered by lymphocyte dysfunction during the inflammatory response, leading to the aggregation of neutrophils and macrophages [63, 64]. Notably, recent studies [19, 20, 65] have discovered that plasma release of mitochondrial DNA (mtDNA), induced by hip fractures, triggers a systemic inflammatory response and lung injury through the activation of the TLR9/NF-KB pathway. Furthermore, intramedullary nailing surgery [25] accelerates the rapid release of mtDNA, exacerbating lung injury in patients with hip fractures and increasing the risk of post-injury lung infection and mortality. This is consistent with the higher incidence of POP observed in patients undergoing intramedullary nailing surgery in our study.

NLR and SII represent the most used biomarkers in clinical practice. They are derived from comprehensive routine tests, do not require special techniques, and offer a cost-effective and easily accessible means of identifying high-risk patients [26, 36]. Furthermore, both NLR and SII are modifiable risk factors that enable physicians to take proactive measures. Researchers are currently exploring advanced strategies for managing POP in hip fracture patients. Recent studies have highlighted the effectiveness of the Enhanced Recovery After Surgery (ERAS) program in reducing postoperative complications among elderly individuals who have undergone hip fracture surgery [66, 67]. The ERAS program has indicated the benefits of various interventions, such as precluding postoperative nausea and vomiting, facilitating early mobilization, engaging in respiratory exercises, and undergoing rehabilitative chest physical therapy for alleviating and modulating POP. Furthermore, other studies have provided evidence on the efficacy of interventions like intensive physical therapy, postoperative pulmonary exercise, and oral care in the prevention and management of POP [48, 68]. Ultimately, the accurate prognosis and identification of POP significantly enhance communication between healthcare providers and patients, particularly when family members are involved.

## Limitations

This study has noteworthy limitations that warrant acknowledgment. Firstly, the retrospective design introduces bias due to the absence of variables and follow-up information. The analyses conducted in this study revealed associations rather than causal relationships. Secondly, since only inpatient data was collected, we were unable to assess the correlation between inflammatory/immune markers and long-term patient follow-up. Thirdly, inflammatory/immune markers may undergo fluctuations during hospitalization. Although efforts were made to mitigate confounding effects by using baseline levels upon admission, changes throughout the entire hospital stay were not analyzed. Lastly, the monitoring of only three biomarkers in a limited sample population necessitates further investigation of additional biomarkers associated with POP in elderly hip fracture patients within a larger cohort.

## Conclusions

The findings of this study demonstrate that the NLR and SII hold predictive value for POP occurrence and adverse outcomes in elderly patients with hip fractures. Among these variables, NLR exhibits the highest predictive performance. It is recommended that healthcare providers remain vigilant for the emergence of POP and undertake proactive measures in cases where the NLR threshold exceeds 5.84 upon admission, thus enabling timely intervention.

### Supplementary Information

**Additional file 1:** Additional materials about this study (including 1. Study flow chart; 2. ROC curves of each biomarker for POP; 3. Subgroup analysis results; 4. Multivariate analysis and propensity score matching results).

## Data Availability

All the data used and analyzed during the current study are available from the corresponding author upon reasonable request.
