# Supplementary material for: Neutrophil-to-lymphocyte ratio (NLR), platelet-to-lymphocyte ratio (PLR), and systemic immune inflammation index (SII) to predict postoperative pneumonia in elderly hip fracture patients
Source: J Orthop Surg Res. 2023 Sep 12;18:673. doi: 10.1186/s13018-023-04157-x (PMC10496383; doi:10.1186/s13018-023-04157-x)
Supplement: Supplementary file 1 — Additional file 1: Additional materials about this study (including 1. Study flow chart; 2. ROC curves of each biomarker for POP; 3. Subgroup analysis results; 4. Multivariate analysis and propensity score matching results). [file 13018_2023_4157_MOESM1_ESM.docx]

**Appendix:**

**eFigure1 Flow diagram of patients included in the cohort.**

**eFigure2 ROC curves of each biomarker for POP.**

**eFigure3 Subgroup analysis of adjustment association between NLR levels and POP After Propensity Score Matching.**

**eFigure4 Subgroup analysis of adjustment association between SII levels and POP After Propensity Score Matching.**

**eTable1 Multivariate Analysis for POP (NLR)**

**eTable2 Multivariate Analysis for POP (PLR)**

**eTable3 Multivariate Analysis for POP (SII)**

**eTable4 Patient Characteristics Before and After Propensity Score Matching by the best cutoff value of NLR**

**eTable5 Patient Characteristics Before and After Propensity Score Matching by the best cutoff value of PLR**

**eTable6 Patient Characteristics Before and After Propensity Score Matching by the best cutoff value of SII**

**
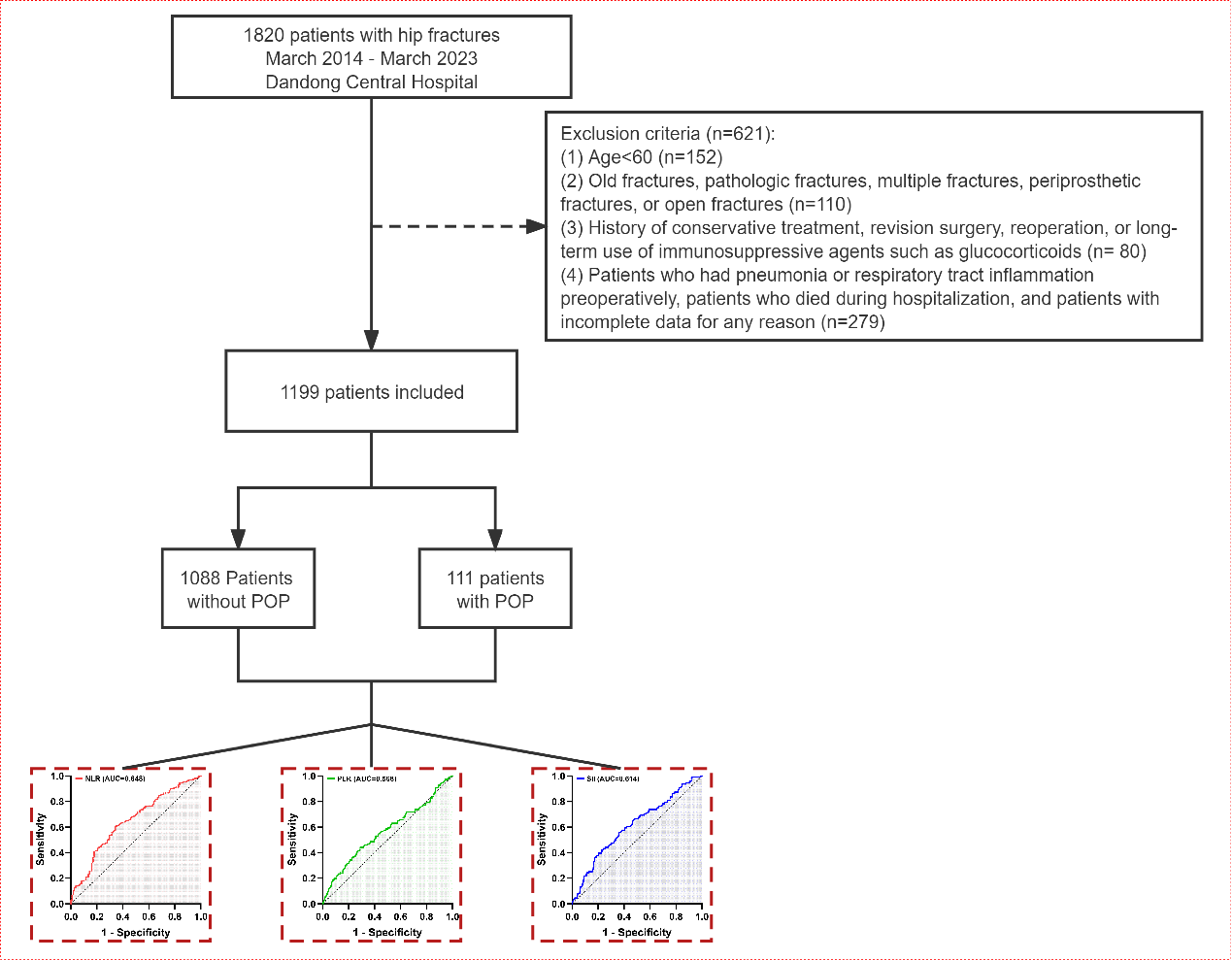
**

**eFigure1 Flow diagram of patients included in the cohort.**


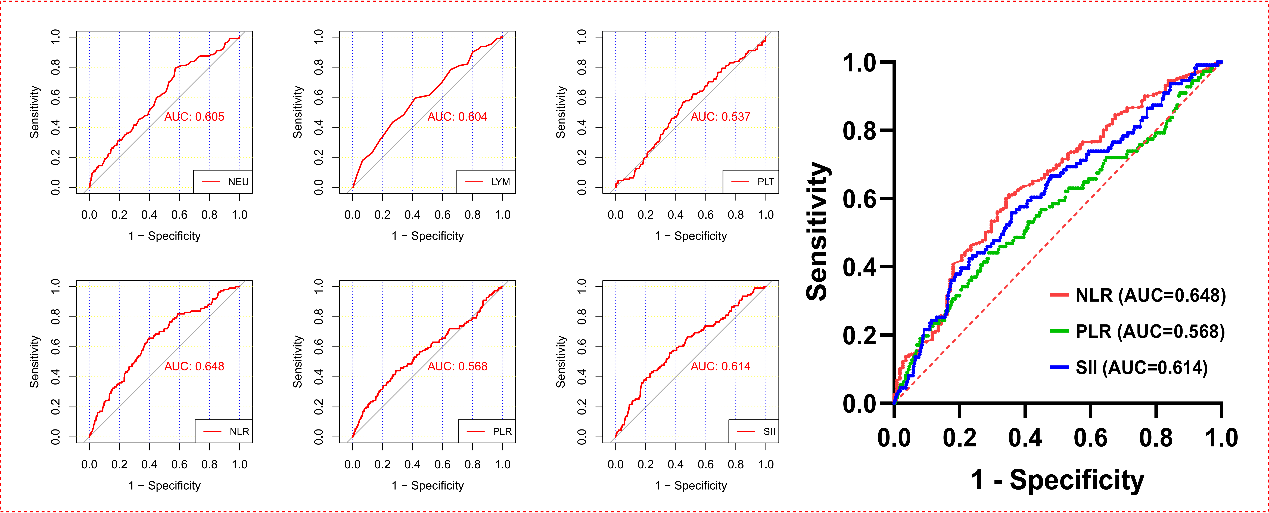


**eFigure2 ROC curves of each biomarker for POP.**


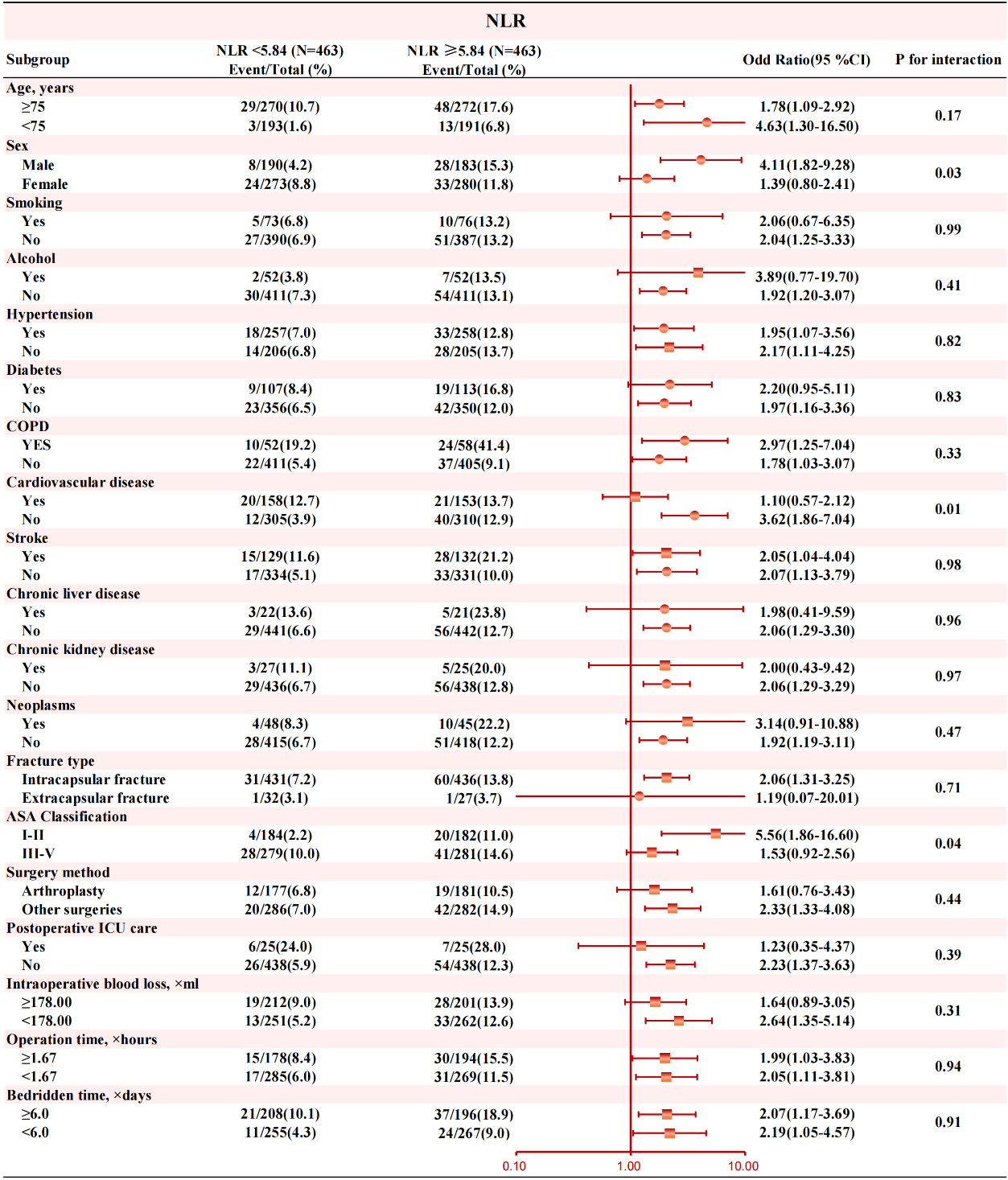


**eFigure3 Subgroup analysis of adjustment association between NLR levels and POP After Propensity Score Matching.**

**
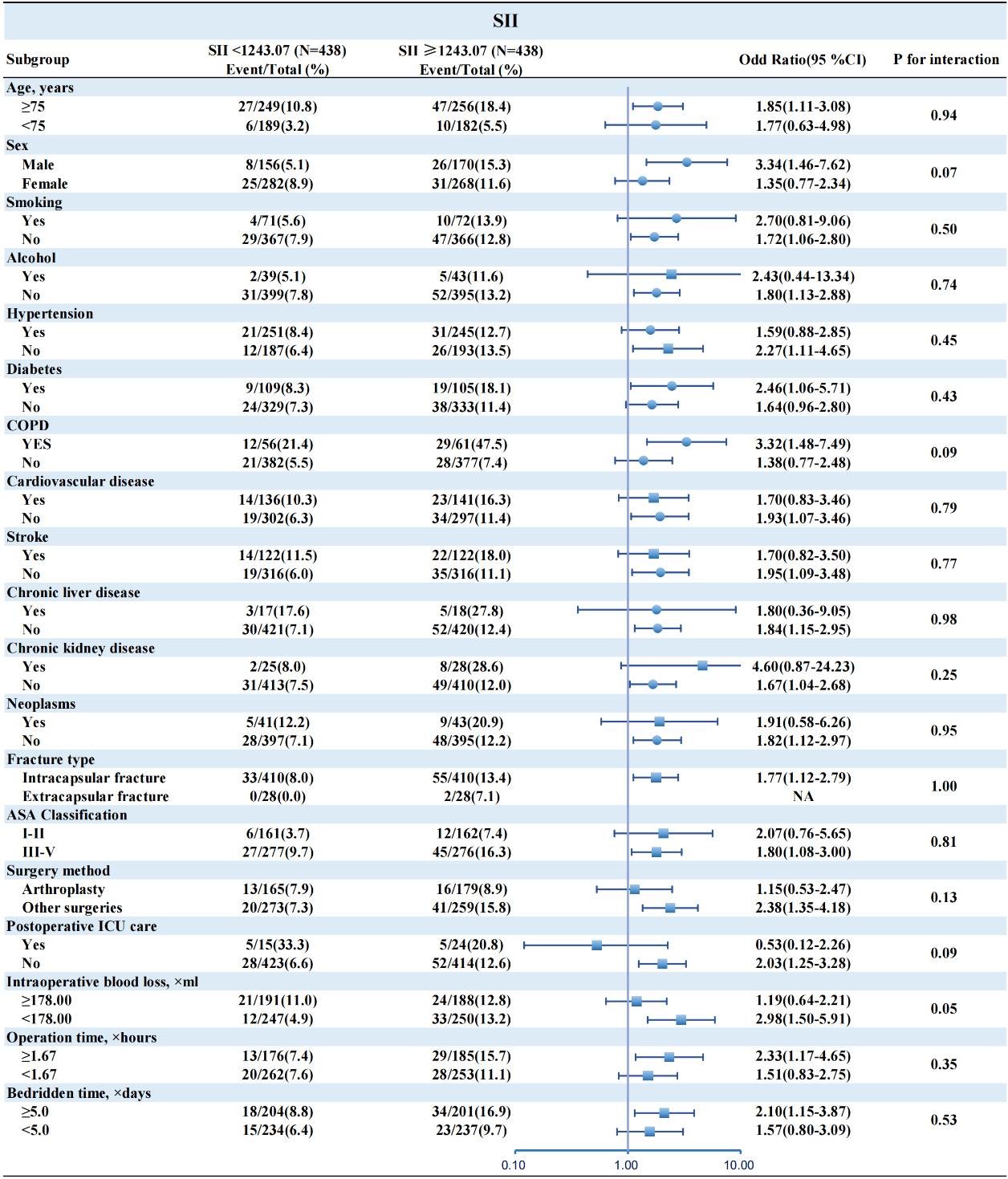
**

**eFigure4 Subgroup analysis of adjustment association between SII levels and POP After Propensity Score Matching. eTable1 Multivariate Analysis for POP (NLR)**

| Characteristics | Univariate | | | Multivariate | | |
| --- | --- | --- | --- | --- | --- | --- |
|  | OR | 95%CI | P-value | OR | 95%CI | P-value |
| Demographic |  |  |  |  |  |  |
| Age, × years | 1.08 | 1.05-1.10 | <0.001 | 1.06 | 1.03-1.08 | <0.001 |
| Female gender | 0.99 | 0.66-1.47 | 0.94 | <NA> | <NA> | <NA> |
| Smoking | 1.00 | 0.60-1.68 | 0.99 | <NA> | <NA> | <NA> |
| Alcohol | 1.04 | 0.57-1.91 | 0.90 | <NA> | <NA> | <NA> |
| Comorbidities |  |  |  |  |  |  |
| Hypertension | 1.40 | 0.94-2.07 | 0.10 | <NA> | <NA> | <NA> |
| Diabetes | 1.41 | 0.91-2.18 | 0.12 | <NA> | <NA> | <NA> |
| COPD | 7.38 | 4.78-11.40 | <0.001 | 5.34 | 3.27-8.71 | <0.001 |
| Cardiovascular disease | 1.87 | 1.26-2.78 | 0.002 | 0.90 | 0.57-1.44 | 0.67 |
| Stroke | 2.50 | 1.68-3.74 | <0.001 | 2.05 | 1.30-3.22 | 0.002 |
| Chronic liver disease | 2.41 | 1.17-4.93 | 0.02 | 1.87 | 0.79-4.46 | 0.16 |
| Chronic kidney disease | 2.52 | 1.30-4.88 | 0.01 | 1.79 | 0.81-4.00 | 0.15 |
| Neoplasms | 1.83 | 1.05-3.19 | 0.03 | 1.27 | 0.65-2.48 | 0.49 |
| Operation |  |  |  |  |  |  |
| Fracture type | 0.70 | 0.51-0.95 | 0.02 | 0.81 | 0.55-1.18 | 0.26 |
| Surgery method | 0.96 | 0.81-1.12 | 0.57 | <NA> | <NA> | <NA> |
| Intraoperative blood loss, ×ml | 1.00 | 0.99-1.00 | 0.43 | <NA> | <NA> | <NA> |
| Postoperative ICU care | 4.74 | 2.59-8.68 | <0.001 | 3.36 | 1.68-6.71 | 0.001 |
| Operation time ×hours | 1.09 | 0.87-1.36 | 0.48 | <NA> | <NA> | <NA> |
| Bedridden time, ×days | 1.11 | 1.07-1.16 | <0.001 | 1.08 | 1.04-1.13 | <0.001 |
| ASA Classification | 2.92 | 1.85-4.60 | <0.001 | 1.32 | 0.78-2.22 | 0.30 |
| Laboratory findings |  |  |  |  |  |  |
| NLR^*^ | 2.92 | 1.94-4.40 | <0.001 | 2.24 | 1.43-3.51 | <0.001 |

*cutoff=5.84, which was identified by Youden’s index.

**eTable2 Multivariate Analysis for POP (PLR)**

| Characteristics | Univariate | | | Multivariate | | |
| --- | --- | --- | --- | --- | --- | --- |
|  | OR | 95%CI | P-value | OR | 95%CI | P-value |
| Demographic |  |  |  |  |  |  |
| Age, × years | 1.08 | 1.05-1.10 | <0.001 | 1.05 | 1.03-1.08 | <0.001 |
| Female gender | 0.99 | 0.66-1.47 | 0.94 | <NA> | <NA> | <NA> |
| Smoking | 1.00 | 0.60-1.68 | 0.99 | <NA> | <NA> | <NA> |
| Alcohol | 1.04 | 0.57-1.91 | 0.90 | <NA> | <NA> | <NA> |
| Comorbidities |  |  |  |  |  |  |
| Hypertension | 1.40 | 0.94-2.07 | 0.10 | <NA> | <NA> | <NA> |
| Diabetes | 1.41 | 0.91-2.18 | 0.12 | <NA> | <NA> | <NA> |
| COPD | 7.38 | 4.78-11.40 | <0.001 | 5.65 | 3.48-9.17 | <0.001 |
| Cardiovascular disease | 1.87 | 1.26-2.78 | 0.002 | 0.93 | 0.58-1.48 | 0.75 |
| Stroke | 2.50 | 1.68-3.74 | <0.001 | 2.03 | 1.29-3.18 | 0.002 |
| Chronic liver disease | 2.41 | 1.17-4.93 | 0.02 | 1.94 | 0.82-4.56 | 0.13 |
| Chronic kidney disease | 2.52 | 1.30-4.88 | 0.01 | 1.90 | 0.86-4.20 | 0.12 |
| Neoplasms | 1.83 | 1.05-3.19 | 0.03 | 1.26 | 0.65-2.42 | 0.50 |
| Operation |  |  |  |  |  |  |
| Fracture type | 0.70 | 0.51-0.95 | 0.02 | 0.79 | 0.54-1.15 | 0.21 |
| Surgery method | 0.96 | 0.81-1.12 | 0.57 | <NA> | <NA> | <NA> |
| Intraoperative blood loss, ×ml | 1.00 | 0.99-1.00 | 0.43 | <NA> | <NA> | <NA> |
| Postoperative ICU care | 4.74 | 2.59-8.68 | <0.001 | 3.33 | 1.69-6.59 | 0.001 |
| Operation time ×hours | 1.09 | 0.87-1.36 | 0.48 | <NA> | <NA> | <NA> |
| Bedridden time, ×days | 1.11 | 1.07-1.16 | <0.001 | 1.08 | 1.03-1.12 | 0.001 |
| ASA Classification | 2.92 | 1.85-4.60 | <0.001 | 1.34 | 0.80-2.25 | 0.27 |
| Laboratory findings |  |  |  |  |  |  |
| PLR^#^ | 1.95 | 1.31-2.90 | 0.001 | 1.37 | 0.88-2.15 | 0.16 |

^#^cutoff=204.41, which was identified by Youden’s index.

**eTable3 Multivariate Analysis for POP (SII)**

| Characteristics | Univariate | | | Multivariate | | |
| --- | --- | --- | --- | --- | --- | --- |
|  | OR | 95%CI | P-value | OR | 95%CI | P-value |
| Demographic |  |  |  |  |  |  |
| Age, × years | 1.08 | 1.05-1.10 | <0.001 | 1.06 | 1.03-1.08 | <0.001 |
| Female gender | 0.99 | 0.66-1.47 | 0.94 | <NA> | <NA> | <NA> |
| Smoking | 1.00 | 0.60-1.68 | 0.99 | <NA> | <NA> | <NA> |
| Alcohol | 1.04 | 0.57-1.91 | 0.90 | <NA> | <NA> | <NA> |
| Comorbidities |  |  |  |  |  |  |
| Hypertension | 1.40 | 0.94-2.07 | 0.10 | <NA> | <NA> | <NA> |
| Diabetes | 1.41 | 0.91-2.18 | 0.12 | <NA> | <NA> | <NA> |
| COPD | 7.38 | 4.78-11.40 | <0.001 | 5.48 | 3.37-8.92 | <0.001 |
| Cardiovascular disease | 1.87 | 1.26-2.78 | 0.002 | 0.94 | 0.59-1.50 | 0.80 |
| Stroke | 2.50 | 1.68-3.74 | <0.001 | 2.07 | 1.32-3.24 | 0.002 |
| Chronic liver disease | 2.41 | 1.17-4.93 | 0.02 | 1.95 | 0.82-4.63 | 0.13 |
| Chronic kidney disease | 2.52 | 1.30-4.88 | 0.01 | 1.88 | 0.85-4.17 | 0.12 |
| Neoplasms | 1.83 | 1.05-3.19 | 0.03 | 1.25 | 0.65-2.43 | 0.50 |
| Operation |  |  |  |  |  |  |
| Fracture type | 0.70 | 0.51-0.95 | 0.02 | 0.79 | 0.54-1.15 | 0.22 |
| Surgery method | 0.96 | 0.81-1.12 | 0.57 | <NA> | <NA> | <NA> |
| Intraoperative blood loss, ×ml | 1.00 | 0.99-1.00 | 0.43 | <NA> | <NA> | <NA> |
| Postoperative ICU care | 4.74 | 2.59-8.68 | <0.001 | 3.40 | 1.71-6.73 | <0.001 |
| Operation time ×hours | 1.09 | 0.87-1.36 | 0.48 | <NA> | <NA> | <NA> |
| Bedridden time, ×days | 1.11 | 1.07-1.16 | <0.001 | 1.08 | 1.03-1.12 | <0.001 |
| ASA Classification | 2.92 | 1.85-4.60 | <0.001 | 1.29 | 0.77-2.18 | 0.33 |
| Laboratory findings |  |  |  |  |  |  |
| SII^&^ | 2.27 | 1.53-3.36 | <0.001 | 1.76 | 1.14-2.73 | 0.01 |

^&^cutoff=1243.07, which was identified by Youden’s index.

**eTable4 Patient Characteristics Before and After Propensity Score Matching by the best cutoff value of NLR**

| Characteristics | Before matching | | | After matching | | |
| --- | --- | --- | --- | --- | --- | --- |
|  | NLR < 5.84  (n=694) | NLR ≥ 5.84  (n=505) | SMD | NLR < 5.84  (n=463) | NLR ≥ 5.84  (n=463) | SMD |
| Demographic |  |  |  |  |  |  |
| Age, × years (Mean, SD) | 73.86 (9.67) | 76.02 (9.39) | 0.23 | 75.87 (9.53) | 75.90 (9.38) | 0.004 |
| Female gender (n, %) | 429 (61.82%) | 291 (57.62%) | 0.09 | 273 (58.96%) | 280 (60.48%) | 0.03 |
| Smoking (n, %) | 119 (17.15%) | 86 (17.03%) | 0.003 | 73 (15.77%) | 76 (16.41%) | 0.02 |
| Alcohol (n, %) | 79 (11.38%) | 57 (11.29%) | 0.003 | 52 (11.23%) | 52 (11.23%) | <0.001 |
| Comorbidities |  |  |  |  |  |  |
| Hypertension (n, %) | 316 (45.53%) | 285 (56.44%) | 0.22 | 257 (55.51%) | 258 (55.72%) | 0.004 |
| Diabetes (n, %) | 148 (21.33%) | 127 (25.15%) | 0.09 | 107 (23.11%) | 113 (24.41%) | 0.03 |
| COPD (n, %) | 57 (8.21%) | 80 (15.84%) | 0.24 | 52 (11.23%) | 58 (12.53%) | 0.04 |
| Cardiovascular disease (n, %) | 195 (28.10%) | 177 (35.05%) | 0.15 | 158 (34.13%) | 153 (33.05%) | 0.02 |
| Stroke (n, %) | 162 (23.34%) | 148 (29.31%) | 0.14 | 129 (27.86%) | 132 (28.51%) | 0.01 |
| Chronic liver disease (n, %) | 27 (3.89%) | 26 (5.15%) | 0.06 | 22 (4.75%) | 21 (4.54%) | 0.01 |
| Chronic kidney disease (n, %) | 28 (4.03%) | 34 (6.73%) | 0.12 | 27 (5.83%) | 25 (5.40%) | 0.02 |
| Neoplasms (n, %) | 66 (9.51%) | 49 (9.70%) | 0.01 | 48 (10.37%) | 45 (9.72%) | 0.02 |
| Operation |  |  |  |  |  |  |
| Fracture type |  |  | 0.14 |  |  | 0.04 |
| Femoral neck fracture (n, %) | 375 (54.03%) | 238 (47.13%) |  | 219 (47.30%) | 225 (48.60%) |  |
| Intertrochanteric fracture (n, %) | 281 (40.49%) | 233 (46.14%) |  | 212 (45.79%) | 211 (45.57%) |  |
| Subtrochanteric fracture (n, %) | 38 (5.48%) | 34 (6.73%) |  | 32 (6.91%) | 27 (5.83%) |  |
| ASA Classification |  |  | 0.28 |  |  | 0.01 |
| Ⅰ-Ⅱ (n, %) | 352 (50.72%) | 187 (37.03%) |  | 184 (39.74%) | 182 (39.31%) |  |
| Ⅲ-V (n, %) | 342 (49.28%) | 318 (62.97%) |  | 279 (60.26%) | 281 (60.69%) |  |
| Surgery method |  |  | 0.36 |  |  | 0.002 |
| Total Hip Arthroplasty (n, %) | 94 (13.54%) | 54 (10.69%) |  | 69 (14.90%) | 51 (11.02%) |  |
| Hemiarthroplasty (n, %) | 146 (21.04%) | 139 (27.52%) |  | 108 (23.33%) | 130 (28.08%) |  |
| Intramedullary nail fixation (n, %) | 223 (32.13%) | 186 (36.83%) |  | 172 (37.15%) | 173 (37.37%) |  |
| Internal fixation with steel plate (n, %) | 86 (12.39%) | 79 (15.64%) |  | 60 (12.96%) | 63 (13.61%) |  |
| Internal fixation with hollow nails (n, %) | 145 (20.89%) | 47 (9.31%) |  | 54 (11.66%) | 46 (9.94%) |  |
| Intraoperative blood loss, ×ml (Mean, SD) | 161.83 (127.25) | 200.49 (182.06) | 0.25 | 181.74 (137.61) | 179.21 (134.24) | 0.02 |
| Postoperative ICU care (n, %) | 28 (4.03%) | 29 (5.74%) | 0.08 | 25 (5.40%) | 25 (5.40%) | <0.001 |
| Operation time, ×hours (Mean, SD) | 1.65 (0.86) | 1.71 (0.74) | 0.07 | 1.67 (0.82) | 1.66 (0.68) | 0.02 |
| Bedridden time, ×days (Mean, SD) | 5.71 (4.08) | 6.15 (3.85) | 0.11 | 5.89 (4.21) | 5.92 (3.60) | 0.01 |

**eTable5 Patient Characteristics Before and After Propensity Score Matching by the best cutoff value of PLR**

| Characteristics | Before matching | | | After matching | | |
| --- | --- | --- | --- | --- | --- | --- |
|  | PLR＜204.41  (n=836) | PLR ≥ 204.41  (n=363) | SMD | PLR＜204.41  (n=357) | PLR ≥ 204.41  (n=357) | SMD |
| Demographic |  |  |  |  |  |  |
| Age, × years (Mean, SD) | 74.00 (9.67) | 76.54 (9.25) | 0.27 | 76.73 (9.71) | 76.40 (9.23) | 0.03 |
| Female gender (n, %) | 494 (59.09%) | 226 (62.26%) | 0.07 | 217 (60.78%) | 222 (62.18%) | 0.03 |
| Smoking (n, %) | 152 (18.18%) | 53 (14.60%) | 0.10 | 52 (14.57%) | 53 (14.85%) | 0.01 |
| Alcohol (n, %) | 99 (11.84%) | 37 (10.19%) | 0.05 | 40 (11.20%) | 37 (10.36%) | 0.03 |
| Comorbidities |  |  |  |  |  |  |
| Hypertension (n, %) | 391 (46.77%) | 210 (57.85%) | 0.22 | 199 (55.74%) | 205 (57.42%) | 0.03 |
| Diabetes (n, %) | 187 (22.37%) | 88 (24.24%) | 0.04 | 92 (25.77%) | 88 (24.65%) | 0.03 |
| COPD (n, %) | 76 (9.09%) | 61 (16.80%) | 0.23 | 48 (13.45%) | 55 (15.41%) | 0.06 |
| Cardiovascular disease (n, %) | 256 (30.62%) | 116 (31.96%) | 0.03 | 124 (34.73%) | 116 (32.49%) | 0.05 |
| Stroke (n, %) | 194 (23.21%) | 116 (31.96%) | 0.20 | 114 (31.93%) | 114 (31.93%) | <0.001 |
| Chronic liver disease (n, %) | 40 (4.78%) | 13 (3.58%) | 0.06 | 8 (2.24%) | 13 (3.64%) | 0.08 |
| Chronic kidney disease (n, %) | 41 (4.90%) | 21 (5.79%) | 0.04 | 21 (5.88%) | 20 (5.60%) | 0.01 |
| Neoplasms (n, %) | 77 (9.21%) | 38 (10.47%) | 0.04 | 36 (10.08%) | 38 (10.64%) | 0.02 |
| Operation |  |  |  |  |  |  |
| Fracture type |  |  | 0.09 |  |  | 0.06 |
| Femoral neck fracture (n, %) | 439 (52.51%) | 174 (47.93%) |  | 165 (46.22%) | 173 (48.46%) |  |
| Intertrochanteric fracture (n, %) | 349 (41.75%) | 165 (45.45%) |  | 166 (46.50%) | 162 (45.38%) |  |
| Subtrochanteric fracture (n, %) | 48 (5.74%) | 24 (6.61%) |  | 26 (7.28%) | 22 (6.16%) |  |
| ASA Classification |  |  | 0.29 |  |  | 0.01 |
| Ⅰ-Ⅱ (n, %) | 412 (49.28%) | 127 (34.99%) |  | 126 (35.29%) | 127 (35.57%) |  |
| Ⅲ-V (n, %) | 424 (50.72%) | 236 (65.01%) |  | 231 (64.71%) | 230 (64.43%) |  |
| Surgery method |  |  | 0.39 |  |  | 0.03 |
| Total Hip Arthroplasty (n, %) | 107 (12.80%) | 41 (11.29%) |  | 58 (16.25%) | 41 (11.48%) |  |
| Hemiarthroplasty (n, %) | 175 (20.93%) | 110 (30.30%) |  | 83 (23.25%) | 108 (30.25%) |  |
| Intramedullary nail fixation (n, %) | 281 (33.61%) | 128 (35.26%) |  | 139 (38.94%) | 127 (35.57%) |  |
| Internal fixation with steel plate (n, %) | 109 (13.04%) | 56 (15.43%) |  | 49 (13.73%) | 53 (14.85%) |  |
| Internal fixation with hollow nails (n, %) | 164 (19.62%) | 28 (7.71%) |  | 28 (7.84%) | 28 (7.84%) |  |
| Intraoperative blood loss, ×ml (Mean, SD) | 171.28 (154.46) | 193.85 (151.54) | 0.15 | 192.50 (167.25) | 191.87 (148.47) | 0.004 |
| Postoperative ICU care (n, %) | 37 (4.43%) | 20 (5.51%) | 0.05 | 20 (5.60%) | 20 (5.60%) | <0.001 |
| Operation time, ×hours (Mean, SD) | 1.64 (0.81) | 1.74 (0.81) | 0.12 | 1.75 (0.92) | 1.73 (0.80) | 0.02 |
| Bedridden time, ×days (Mean, SD) | 5.67 (3.86) | 6.43 (4.22) | 0.19 | 6.40 (4.73) | 6.34 (4.16) | 0.01 |

**eTable6 Patient Characteristics Before and After Propensity Score Matching by the best cutoff value of SII**

| Characteristics | Before matching | | | After matching | | |
| --- | --- | --- | --- | --- | --- | --- |
|  | SII＜1243.07  (n=747) | SII ≥ 1243.07  (n=452) | SMD | SII＜1243.07  (n=438) | SII ≥ 1243.07  (n=438) | SMD |
| Demographic |  |  |  |  |  |  |
| Age, × years (Mean, SD) | 74.22 (9.60) | 75.68 (9.58) | 0.15 | 75.42 (9.23) | 75.68 (9.61) | 0.03 |
| Female gender (n, %) | 447 (59.84%) | 273 (60.40%) | 0.01 | 282 (64.38%) | 268 (61.19%) | 0.07 |
| Smoking (n, %) | 126 (16.87%) | 79 (17.48%) | 0.02 | 71 (16.21%) | 72 (16.44%) | 0.01 |
| Alcohol (n, %) | 91 (12.18%) | 45 (9.96%) | 0.07 | 39 (8.90%) | 43 (9.82%) | 0.03 |
| Comorbidities |  |  |  |  |  |  |
| Hypertension (n, %) | 348 (46.59%) | 253 (55.97%) | 0.19 | 251 (57.31%) | 245 (55.94%) | 0.03 |
| Diabetes (n, %) | 166 (22.22%) | 109 (24.12%) | 0.05 | 109 (24.89%) | 105 (23.97%) | 0.02 |
| COPD (n, %) | 64 (8.57%) | 73 (16.15%) | 0.23 | 56 (12.79%) | 61 (13.93%) | 0.03 |
| Cardiovascular disease (n, %) | 227 (30.39%) | 145 (32.08%) | 0.04 | 136 (31.05%) | 141 (32.19%) | 0.03 |
| Stroke (n, %) | 181 (24.23%) | 129 (28.54%) | 0.10 | 122 (27.85%) | 122 (27.85%) | <0.001 |
| Chronic liver disease (n, %) | 35 (4.69%) | 18 (3.98%) | 0.04 | 17 (3.88%) | 18 (4.11%) | 0.01 |
| Chronic kidney disease (n, %) | 34 (4.55%) | 28 (6.19%) | 0.07 | 25 (5.71%) | 28 (6.39%) | 0.03 |
| Neoplasms (n, %) | 71 (9.50%) | 44 (9.73%) | 0.01 | 41 (9.36%) | 43 (9.82%) | 0.02 |
| Operation |  |  |  |  |  |  |
| Fracture type |  |  | 0.09 |  |  | 0.02 |
| Femoral neck fracture (n, %) | 392 (52.48%) | 221 (48.89%) |  | 212 (48.40%) | 217 (49.54%) |  |
| Intertrochanteric fracture (n, %) | 315 (42.17%) | 199 (44.03%) |  | 198 (45.21%) | 193 (44.06%) |  |
| Subtrochanteric fracture (n, %) | 40 (5.35%) | 32 (7.08%) |  | 28 (6.39%) | 28 (6.39%) |  |
| ASA Classification |  |  | 0.29 |  |  | 0.01 |
| Ⅰ-Ⅱ (n, %) | 376 (50.33%) | 163 (36.06%) |  | 161 (36.76%) | 162 (36.99%) |  |
| Ⅲ-V (n, %) | 371 (49.67%) | 289 (63.94%) |  | 277 (63.24%) | 276 (63.01%) |  |
| Surgery method |  |  | 0.39 |  |  | 0.004 |
| Total Hip Arthroplasty (n, %) | 98 (13.12%) | 50 (11.06%) |  | 61 (13.93%) | 49 (11.19%) |  |
| Hemiarthroplasty (n, %) | 152 (20.35%) | 133 (29.42%) |  | 104 (23.74%) | 130 (29.68%) |  |
| Intramedullary nail fixation (n, %) | 259 (34.67%) | 150 (33.19%) |  | 169 (38.58%) | 147 (33.56%) |  |
| Internal fixation with steel plate (n, %) | 87 (11.65%) | 78 (17.26%) |  | 55 (12.56%) | 71 (16.21%) |  |
| Internal fixation with hollow nails (n, %) | 151 (20.21%) | 41 (9.07%) |  | 49 (11.19%) | 41 (9.36%) |  |
| Intraoperative blood loss, ×ml (Mean, SD) | 167.07 (136.69) | 196.37 (177.34) | 0.19 | 184.24 (152.97) | 185.64 (155.11) | 0.01 |
| Postoperative ICU care (n, %) | 32 (4.28%) | 25 (5.53%) | 0.06 | 15 (3.42%) | 24 (5.48%) | 0.10 |
| Operation time, ×hours (Mean, SD) | 1.65 (0.85) | 1.71 (0.75) | 0.09 | 1.71 (0.83) | 1.70 (0.73) | 0.01 |
| Bedridden time, ×days (Mean, SD) | 5.61 (3.88) | 6.37 (4.12) | 0.19 | 6.19 (4.41) | 6.21 (3.91) | 0.01 |
